# Nanoencapsulation of Biotics: Feasibility to Enhance Stability and Delivery for Improved Gut Health

**DOI:** 10.3390/pharmaceutics17091180

**Published:** 2025-09-11

**Authors:** Pedro Brivaldo Viana da Silva, Thiécla Katiane Osvaldt Rosales, João Paulo Fabi

**Affiliations:** 1 Department of Food Science and Experimental Nutrition, School of Pharmaceutical Sciences, University of São Paulo, São Paulo 05508-220, SP, Brazil; pedroviana@usp.br (P.B.V.d.S.); thieclarosales@usp.br (T.K.O.R.); 2 Food Research Center (FoRC), CEPID-FAPESP (Research, Innovation and Dissemination Centers, São Paulo Research Foundation), CEPIX-USP, São Paulo 05508-080, SP, Brazil; 3 Food and Nutrition Research Center (NAPAN), University of São Paulo, São Paulo 05508-220, SP, Brazil

**Keywords:** gut microbiota, probiotics, prebiotics, synbiotics, postbiotics, nanoencapsulation

## Abstract

The human gastrointestinal tract contains a complex and diverse community of microorganisms, referred to as the gut microbiota. Due to their close proximity to human cells, these microorganisms play a crucial role in maintaining the host’s health, influencing various metabolic processes, and providing protection against potentially harmful agents and pathogens. The disruption in this microbial ecosystem, known as dysbiosis, is associated with inflammatory and metabolic diseases, as well as certain types of cancer. Strategies to modulate the microbiota toward a state of homeostasis through the use of “biotics” (probiotics, prebiotics, synbiotics, and postbiotics) have increased. However, challenges such as low stability, loss of microbial viability, and difficulties in delivery to the intestine significantly decrease the progress of their clinical and nutritional applications. Microencapsulation and nanoencapsulation technologies offer potential solutions to enhance the stability, bioavailability, and controlled release of microorganisms and/or bioactive compounds within the gastrointestinal tract. Considering these aspects, this review provides a comprehensive overview of recent advances in nanoencapsulation techniques for biotics, highlighting their mechanisms of action, potential health benefits, and applications in functional foods and targeted therapies. Furthermore, it addresses existing limitations, evaluates feasibility, and discusses the future potential of these technologies in promoting gut health and disease prevention. Further research, especially through clinical studies, is mandatory to verify the safety and effectiveness of nanoencapsulated biotics and to obtain regulatory approval.

## 1. Introduction

The human intestine harbors a complex ecosystem of microorganisms that colonize all its segments, and the connection between these microbes and human cells has profound effects on health [1,2]. The gut-resident microbiota significantly influences the digestion and absorption of nutrients, modulates intestinal endocrine function, synthesizes vitamins, supports the elimination of pathogens and xenobiotics, and may potentially impact inflammation and the development of colon carcinogenesis [3]. It is essential to recognize that how the microbiota exerts these effects is highly dependent on its state. Whether in a state of homeostasis or dysbiosis, each condition distinctly influences the microbiota’s functional capacity and, consequently, its impact on the host’s physiology [4].

The primary definition of intestinal microbiota homeostasis is the absence of diagnosed gastrointestinal diseases or disorders, or the lack of digestive symptoms without specific diagnoses [5]. This definition is clinically useful, but it overlooks subclinical conditions or imbalances that are not manifested as diagnosable diseases. To circumvent this issue, efforts have been made regarding the ecology of a community in homeostasis, its functional core, and perspectives such as resistance, resilience, microbial stability, and the production of metabolites [6,7]. On the other hand, multiple factors, including genetic predisposition, diet, medication use, infections, stress, and medical interventions, can destabilize this intestinal ecosystem, leading to dysbiosis [8,9]. Dysbiosis, a phenomenon characterized by disruption in the intestinal ecosystem, is accompanied by various events, notably a decrease in microbial diversity, particularly the reduction in beneficial microorganisms such as those producing short-chain fatty acids (SCFAs) (*Faecalibacterium*, *Roseburia*, *Lachnospiraceae*, and *Eubacterium*), increased mucosal layer degradation, and expansion of pathogenic microorganisms, such as Gram-negative bacteria, thereby increasing lipopolysaccharide (LPS) content [10]. These microbiota disturbances have been linked to the development of various conditions, including inflammatory bowel diseases [11], metabolic disorders [12], allergies [13], and even neoplasms [14].

Correcting dysbiosis may help prevent or treat diseases and alleviate symptoms [2,15], and this can be achieved by correctly modulating the ecosystem to restore balance and promote health. Among these approaches, ‘biotics’—a comprehensive category encompassing probiotics, prebiotics, synbiotics, and postbiotics—has gained notoriety [16].

Despite the broad therapeutic potential of biotics, their practical application faces significant challenges. In the case of probiotics, for instance, the viability of microorganisms can be severely compromised during processing, storage, and particularly in the gastrointestinal tract, due to exposure to the acidic pH of the stomach, digestive enzymes, bile salts, and oxygen [17,18]. Prebiotics, on the other hand, may undergo rapid fermentation in the most proximal part of the intestine, causing gas distension (bloating) and limiting their effective action in the colon [19].

Encapsulation processes offer the potential to explore the biological use of new bioactives and active molecules for human benefit, since the technology provides physical protection, controlled release, and increased stability and bioavailability of the encapsulates. In the case of pro- and prebiotics, encapsulation methodologies typically rely on microparticles, as exemplified by the work of Yin et al. [20], who encapsulated the probiotic *Escherichia coli* Nissle 1917 (EcN) within prebiotics (alginate and inulin). This approach enhanced the system stability during digestion and demonstrates potential as an adjunctive therapy in treating inflammatory bowel disease.

However, the imperfections on the microparticles, especially thick and uneven surfaces, can lead to changes in the sensorial quality of foods and supplements, resulting in low acceptability. Larger particles can also alter the release mechanisms in the gastrointestinal tract, leading to imprecise delivery [21]. In response to these challenges, the use of nanotechnology has gained attention for biotic delivery.

The primary aim of this article is to provide an understanding of the technologies associated with “biotics” delivery systems based on the physiological characteristics of the gastrointestinal tract. The discussion encompasses key advancements, as well as the feasibility, limitations, and prospects within this field, with a focus on promoting intestinal health and disease prevention.

## 2. Biotics: Main Characteristics and Bioactivity

**Figure 1:**
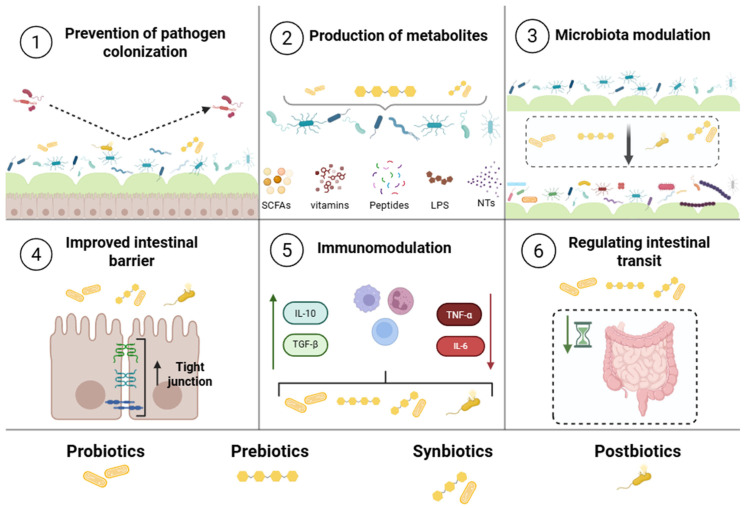
The main mechanisms of how biotics work, as outlined by the International Scientific Association for Probiotics and Prebiotics (ISAPP).

The adjective “biotic”, as defined by the Oxford English Dictionary, refers to something that is related to or resulting from living organisms. In the field of Biology, this term is used to describe a wide range of contexts involving living organisms, their interactions with other living beings, and with the environment. In the context of this text, “biotics” encompasses living and non-living microorganisms, substrates, and their combinations, which can interact with the microbial community of the gastrointestinal tract—particularly in its lower portion—thereby providing benefits to the host. Examples of such elements include probiotics, prebiotics, synbiotics, and postbiotics (Figure 1).

Each type of biotic has already had its definition reviewed by the International Scientific Association for Probiotics and Prebiotics (ISAPP). Probiotics are defined as live microorganisms that, when administered in adequate amounts, confer a health benefit on the host. This definition emphasizes the importance of the viability of microorganisms and requires that, for microbial species to be recognized as probiotics, their health benefits must be properly demonstrated [22]. In contrast, the definition of postbiotics includes preparations of inanimate microorganisms and/or their components that confer a health benefit on the host [23]. This definition does not require that the original microorganism be considered a probiotic; nevertheless, it does require characterization of the strain used and evidence of its benefits. Furthermore, this definition excludes vaccines, purified metabolites, and viral products from the definition of postbiotics.

Only a limited number of microorganisms can be classified as probiotics. Among these, the genera *Lactobacillus* and *Bifidobacterium* are regarded as classic examples, having been extensively studied due to their diverse origins and mechanisms of action [24]. Common sources of probiotics include human or other animal milk, feces, as well as traditional and non-traditional fermented foods. These microorganisms can subsequently be used as dietary supplements or as starter cultures for fermented foods [25,26]. Advances in sequencing technologies and comparative analyses of the microbiota have enabled the exploration of microorganisms belonging to genera with no previous history of probiotic use but possessing novel properties. Notable examples include *Roseburia intestinalis*, *Eubacterium* spp., *Akkermansia muciniphila*, *Faecalibacterium prausnitzii*, and *Bacteroides* spp. [27,28]. The mechanisms of action of probiotics are varied and include preventing pathogen colonization through competitive exclusion; producing antimicrobial metabolites; modulating the intestinal microbiota; strengthening the intestinal barrier; immunomodulating; metabolizing bioactive molecules, such as bacteriocins; synthesizing essential nutrients; and regulating intestinal transit [22]. Notably, these biological effects are not limited to the gut but may also extend to other organ systems, underscoring the potential use of probiotics as adjuncts in the prevention and treatment of various clinical conditions, including colitis [29], Alzheimer’s disease [30], and atopic dermatitis [31]. However, technical challenges in ensuring the survival of these organisms throughout the production chain of functional foods have driven an increasing interest in the concept of postbiotics [32].

Postbiotics comprise a range of biologically active components, including short-chain fatty acids, exopolysaccharides, enzymes, peptides, teichoic acids, and cell wall fragments, which exhibit antimicrobial, anticancer, and immunomodulatory activities [33]. The mechanisms of action of postbiotics include modulation of the immune response, strengthening of the intestinal barrier, inhibition of pathogen colonization, and reduction in inflammation [23]. Improvements in symptoms of irritable bowel syndrome (IBS) were observed with *Bifidobacterium longum* CECT 7347 and its respective postbiotic in a randomized, double-blind clinical trial [34]. The results indicated that both treatments reduced IBS symptoms, including abdominal pain, abdominal distension, satisfaction with bowel habits, stool consistency, and anxiety. Postbiotics demonstrated effects comparable to those of the probiotic, with the advantage of requiring less stability maintenance, as they do not contain live microorganisms.

The definitions of probiotics and postbiotics do not mention the involvement of the microbiota as a mechanistic basis for their benefits [16]. Nevertheless, the interaction with the microbiota—and thus its composition—is one of the main biological foundations for most of the positive effects observed with the consumption of probiotics and postbiotics. Nonetheless, this omission tends to render the definitions broader, as beneficial effects may also arise through other mechanisms, such as immune modulation, enhancement of the epithelial barrier, metabolic modulation, and signaling via the nervous system.

Prebiotics and synbiotics are fundamentally based on the mediation of their beneficial effects through the metabolism of microorganisms within the intestinal microbiota. The ISAPP defines prebiotics as a substrate that is selectively utilized by host microorganisms, conferring a health benefit [35]. Similarly, synbiotics are characterized as a combination consisting of live microorganisms and substrate(s) selectively utilized by host microorganisms, which provide a health benefit to the host [36]. Thus, both prebiotics and synbiotics rely on the metabolic activity of the microbiota for their transformation and the attainment of their intended effects.

The definition of prebiotics proposed by ISAPP expands this classification beyond carbohydrates that cannot be digested and absorbed by the human body (fermentable dietary fibers and oligosaccharides), also including substances such as (poly)phenols [37] and polyunsaturated fatty acids [38,39]. Although nanoencapsulation methods for polyphenols and unsaturated fatty acids are well-understood, the emerging benefits of carbohydrate nanoencapsulation are gaining attention and will be explored in this review.

The gut microbiota degrades and metabolizes prebiotics, stimulating the growth and activity of specific bacterial communities producing diverse beneficial metabolites, potentially inducing positive host metabolic changes [40]. Thus, the main mechanisms of action of prebiotics derive from the production of metabolites, such as short-chain fatty acids, which may exert localized effects (such as improving the intestinal barrier and reducing intestinal pH) and/or systemic effects (such as regulating lipid and glucose metabolism) [35]. Prebiotics can also directly interact with host cells, for example, by modulating the immune response through the activation of toll-like receptors. This effect may even be used as an adjuvant in the treatment of cancer with anti-PD1/PD-L1 monoclonal antibody immunotherapy, in lung cancer [41], breast cancer [42], and melanoma [43].

The combination of live microorganisms and substrates utilized by the microbiota represents an approach to enhance the beneficial effects on the intestinal ecosystem. This is the concept of synbiotics. In addition to combining the individual impact of probiotics and prebiotics, their joint action may generate superior or distinct benefits, as prebiotics provide a favorable environment for the survival and activity of probiotic microorganisms [44]. The most common synbiotic combinations include *Lactobacillus* and *Bifidobacterium* as probiotics, together with FOS or inulin as prebiotics [45]. The combination of *Pediococcus acidilactici* CECT9879, as the microorganism, and oat β-glucans, as a substrate for the microbiota, achieved beneficial modulation of glucose metabolism through different mechanisms, including modulation of the microbiota and the overexpression of Glut-1 and Glut-4 in adipose and muscle tissues [46]. The results were shown to be superior to the effects observed with the individual components.

Oral consumption is the usual form of biotic intake. Nevertheless, this method also presents significant challenges, depending on the expected effects of the biotic. Microbial cells must survive through the gastrointestinal tract while maintaining their viability to exhibit their bioactive properties. The gastrointestinal environment presents various challenges, including the presence of lysozyme in the oral mucosa, the acidic environment of gastric juice, and the enzymatic complexes in the intestine. On the other hand, although oligosaccharides commonly exhibit significant resistance to human enzymes in the upper gastrointestinal tract and stability against pH changes, their fermentation occurs rather rapidly in the initial portions of the colon, which limits their activity in more distal regions of the colon [21].

## 3. Nanoencapsulation

The classical definition of nanomaterials is based on their dimensions, although the use is frequently context-dependent. Most regulations describe nanomaterials as objects with dimensions between 1 and 100 nm. This convention has broad acceptance in chemical sciences. However, due to the commonly polydisperse nature of nanomaterials, the European Union Observatory for Nanomaterials has considered nanomaterials in which at least 50% of the particles fall within the 1–100 nm range [47]. Nevertheless, some definitions, such as those in the pharmaceutical and food field, consider nanometric systems to include particles with sizes ranging from several nanometers to below 1000 nm [48], because a significant portion of particles still exists within the 1–100 nm range, and their physicochemical behavior differs notably from that of micrometer-scale materials. Since this review mainly focuses on applications in the food sector—while also covering pharmaceutical and medical studies, especially oral delivery—we use the standard terminology of referring to “nanoparticles” as systems with particle size distributions ranging from 1 to 1000 nm.

Nanometric-scale dimensions impart physical and chemical properties that are significantly distinct from those observed in their micrometric or bulk counterparts. These differences are evident in characteristics such as color, strength, conductivity, and reactivity. The reduced size and unique structure of nanomaterials are indicated for specific applications where micromaterials are ineffective [49]. An example is the surface properties of nanomaterials, which arise from their high specific surface area and the greater number of atoms located at the surface. Furthermore, the lower coordination of these atoms—that is, the reduced number of direct neighbors—contributes to increased surface reactivity [50].

Returning to the protection of sensitive compounds, nanoencapsulation establishes a physicochemical barrier between the core—viable cells and/or bioactive compounds—and the external milieu [48]. This barrier, commonly referred to as the wall, matrix, or encapsulating agent, is typically constructed from food-grade materials such as proteins, polysaccharides, lipids, or their hybrids, reflecting the prominence of systems designed for oral delivery. The benefits conferred by nanoencapsulation depend on the needs of the core: enhanced apparent solubility and sustained dispersion in aqueous media for hydrophobic compounds; modulation of release along the gastrointestinal tract, enabling site-specific delivery; and protection against degradative stressors, including extreme pH, light, oxygen, digestive enzymes, and bile salts [51]. Depending on the encapsulating material, additional functionalities may be engineered to improve further performance, such as mucoadhesion and the facilitation of transcellular or paracellular transport [52]. From an industrial processing perspective, nanoencapsulation enhances the stability of thermolabile compounds, masks undesirable flavors, protects against oxidation, and can enable milder preservation conditions [53].

Over the past decade, research on the benefits of orally delivered nanocapsules has become increasingly prevalent. Numerous encapsulation techniques and polymeric materials have already been explored, with a particular emphasis on protection against the adverse conditions of different sections of the gastrointestinal tract or on targeted release at specific sites [48].

Nanoencapsulation systems can be fabricated using a range of technologies grounded in top–down or bottom–up strategies, including emulsification, polymeric coating, complex coacervation, nanoprecipitation, and complexation [54]. The use of non-toxic, biocompatible, and renewable shell materials offers clear advantages—reducing environmental impact and minimizing hazardous waste. Nevertheless, the compositional and structural complexity of these food-grade matrices often hampers tight control over nanoparticle size, morphology, and properties when compared with syntheses employing synthetic materials and well-established chemical routes [55]. Recently, microfluidics-based manufacturing has gained prominence owing to its high reproducibility, low batch-to-batch variation, superior control over particle attributes, and straightforward scalability to larger-scale or continuous production [56].

To understand the main topics in research on the nanoencapsulation of biotics, a bibliometric study was conducted using bibliometric data obtained from the Science Citation Index Expanded—Web of Science (WoS) core collection by Clarivate Analytics. An advanced search was performed using keywords, targeting terms present only in the title (“TI”), abstract (“AB”), or author keywords (“AK”) of the documents. For terms related to nanoencapsulation, the following descriptors were used: “nanoencapsulat*”, “nano-encapsulat*”, “nanocarrier*”, “nanoparticle*”, “nanocapsule*”, and “nanostructur*”. The “biotics” categories were based on the definitions established by the International Scientific Association for Probiotics and Prebiotics (ISAPP) to date (“probiotic*”, “prebiotic*”, “synbiotic*”, and “postbiotic*”). The asterisk (*) truncation character was applied to all terms to capture different spelling variants. The results were filtered by document type (“Article” and “Review”) and language (English). The search yielded a total of 841 articles, and the data were exported and analyzed using VOSviewer (version 1.6.20).

**Figure 2:**
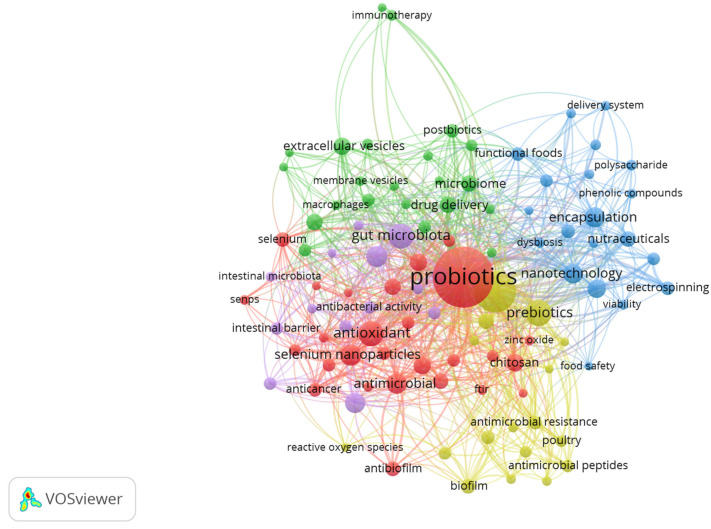
VOSviewer map from the search combination of the terms “Biotics” and “Nanoencapsulation”.

Figure 2 presents a co-occurrence graph of the most frequently used keywords by authors in the studies resulting from the bibliometric search. The importance of research on probiotics is evident from its central position in the graph, as well as its numerous connections to other keywords. Research on probiotics is described by the red cluster, exemplified by other keywords such as “*Lactobacillus*”, “*Lactobacillus acidophilus*”, “*Lactobacillus rhamnosus*”, “lactic acid bacteria”, and “bacteriocins”. Keywords such as “antioxidant”, “antimicrobial”, “anticancer”, and “antibacterial” are interconnected, indicating a strong association of biotics with antioxidant and antimicrobial effects. The green cluster represents research at the intersection of biotics, gut microbiota, immune system, and signaling, exemplified by terms such as “gut microbiota”, “microbiome”, “macrophages”, “extracellular vesicles”, “immunotherapy”, “membrane vesicles”, “anti-inflammatory”, and “immunomodulation”. The blue cluster forms a thematic axis focused on the development of innovative systems for the delivery and stabilization of biotics, exemplified by terms such as “electrospinning”, “nanotechnology”, “nanofiber”, “controlled release”, and “stability”. “Functional foods”, “nutraceuticals”, and “delivery system” point to practical applications in the food and health industries. The purple cluster bridges technology, therapeutics, and functionality, encompassing topics such as “nanomaterials”, “biosynthesis”, “inflammatory bowel disease”, “ulcerative colitis”, and “intestinal barrier”. Finally, the yellow cluster focuses on the practical applications of nanoencapsulated biotics, particularly in controlling microbial growth and antimicrobial resistance, as exemplified by terms such as “antimicrobial resistance”, “antibiotic resistance”, “antibiotics”, “biofilm”, “quorum sensing”, and “antimicrobial peptides”. The topics addressed here extend beyond human health, encompassing applications in veterinary medicine and food safety.

## 4. Nanoencapsulation of Biotics

### 4.1. Single-Cell Nanocoating of Probiotics

Although the use of more resistant probiotic strains has become increasingly common in recent years, these microorganisms are still subjected to numerous unfavorable factors—such as variations in pH, heat, light, and adverse conditions in the gastrointestinal tract (temperature, pH, and oxygen concentration)—from formulation until delivery to the target site within the gastrointestinal tract. This exposure may consequently reduce their viability to levels below the optimal range (10^8^ to 10^9^ CFU) necessary for achieving health benefits [57]. Therefore, encapsulation strategies represent crucial measures to enhance the technical viability of approaches involving the oral administration of microorganisms.

A key point of discussion in nanoencapsulation processes is the dimensions of the systems employed. Probiotic cells typically range in size from 1 to 10 µm, clearly exceeding the dimensions conventionally defined for nanostructured systems. Consequently, the most common encapsulation approaches for probiotics have primarily utilized microencapsulation techniques, in which microbial cells are embedded within a matrix, commonly through extrusion, emulsification, and spray-drying techniques [18]. While these approaches have demonstrated some success in enhancing probiotic viability, critical limitations, such as uncontrolled particle size, leakage, and low in vivo efficacy, continue to pose significant challenges [58]. Furthermore, the relatively large dimensions of probiotic-containing particles present sensorial issues when incorporated into food products, thereby reducing their consumer appeal.

**Figure 3:**
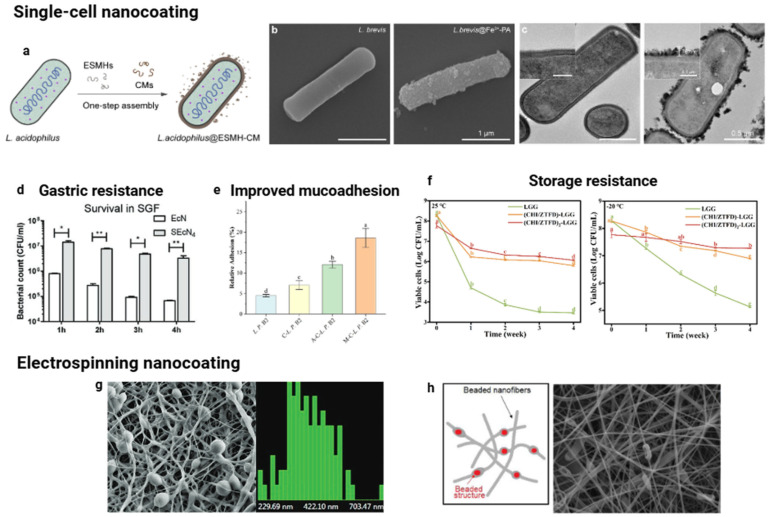
Single-cell nanocoating technologies for protecting probiotic cells. (**a**) Schematic representation of the formation of nanocoatings on individual *Lactobacillus acidophilus* cells. Reproduced from [60], licensed under CC BY 4.0. (**b**,**c**) Scanning and transmission electron microscopy images of *Levilactobacillus brevis* without and with Fe^3+^-phytic acid nanoshells, respectively. Adapted with permission from Ref. [64]. Copyright (2025) American Chemical Society. (**d**) Number of surviving free and coated *Escherichia coli* Nissle 1917 cells after exposure to gastric conditions (Statistical differences: * *p* < 0.05 and ** *p* < 0.01). Adapted with permission from Ref. [61]. Copyright (2021) John Wiley and Sons. (**e**) Relative adhesion rate of *Lactiplantibacillus plantarum* B2 under simulated intestinal conditions (Different letters in (**e**,**f**) indicate significant differences (*p* < 0.05)). Adapted with permission from Ref. [63]. Copyright (2025) Elsevier. (**f**) Viability of *Lacticaseibacillus rhamnosus* GG after four weeks of storage at 25 °C or 20 °C. Adapted with permission from Ref. [62]. Copyright (2024) Elsevier. (**g**) Scanning images and corresponding size distribution histograms of nanofibers containing *L. acidophilus* derived from okara. Adapted with permission from Ref. [65]. Copyright (2011) American Chemical Society. (**h**) Nanofiber mats loaded with *Lactobacillus paracasei* KS-199 (Scale bar = 2 µm). Adapted with permission from Ref. [66]. Copyright (2020) Elsevier.

To address these challenges, novel encapsulation technologies are currently under development. For instance, the creation of an additional membrane surrounding probiotic cells through biomaterials, known as single-cell technology, exemplifies this advancement (Figure 3a–f). This technology utilizes functional groups present on bacterial surfaces, such as sulfhydryl, hydroxyl, carboxyl, and amino groups, to facilitate binding with nanoencapsulating materials—including peptidoglycan, teichoic acid, lipoproteins, lipids, and proteins—thereby forming nanoscale coatings (nanocoating) [59]. For example, Han et al. [60] explored the use of biomolecules derived from food residues, specifically eggshell membrane hydrolysates and coffee melanoidins, as coatings for *Lactobacillus acidophilus*, resulting in no perceptible reduction in viability and offering protection against gastric fluids.

Enhancing the preservation of probiotic cells can be accomplished by augmenting the thickness of the surrounding matrix nanocoating. Hou et al. [61] demonstrated that *Escherichia coli* Nissle 1917 exhibited improved survival in gastric conditions as the number of nanocoating cycles increased. The improvement in viability was seen without reducing its ability to grow. A similar effect was observed with the encapsulation of *Lacticaseibacillus rhamnosus* GG, wherein the multiple nanocoating layers markedly enhanced cell survival during freezing and high-temperature stress [62]. Furthermore, this multilayered approach yielded enhanced in vivo viability, signifying that the reinforced barrier effectively mitigated the adverse effects of gastric acid and bile salts. The combination of different compounds in the barrier can result in better performance compared to using a single compound. *Lactobacillus plantarum* B2, nanocoated with chitosan, demonstrated enhanced resistance to gastrointestinal conditions, particularly when layered with sodium alginate or mucin [63]. Moreover, these extra layers significantly improved cell viability during storage and oxidative stress. Notably, mucin nanocoating effectively increased the adhesion efficiency of *L. plantarum* B2 to the intestinal mucosa. Additionally, the bilayer did not impede cell growth or the probiotic’s production capacity during fermentation, making it a viable option for addressing riboflavin deficiency.

Various challenges faced by probiotics in the gastrointestinal environment, including enzymatic attacks and antibiotic exposure, can be mitigated by the single-cell nanocoating technique. A protocol based on a biphasic water–oil system was used for the individual cell coating of *Lactobacillus acidophilus* [67]. By vortexing, a supramolecular self-organized interface composed of luteolin and Fe^3+^ ions was formed directly on the probiotic cell surface. This nanocoating conferred high resistance against bacterial lysis induced by lysozyme, maintaining approximately 95% cell viability, in contrast to nearly 100% lysis observed in non-encapsulated cells. Additionally, nanocoatings based on tannic acid-Fe^3+^ complexes have demonstrated effective protection against antibiotics commonly used in clinical practice [68]. Probiotics encapsulated in these nanoshells showed strong resistance to multiple antibiotic classes, including ciprofloxacin, tobramycin, gentamicin, and levofloxacin, in both laboratory and animal studies, thereby helping to prevent dysbiosis and reduce symptoms of antibiotic-associated diarrhea. Furthermore, another investigation utilizing nanoshells constructed from Fe^3+^-phytic acid, approximately 80 nm in thickness, also evidenced substantial safeguarding of *Levilactobacillus brevis* against adverse conditions, including gastric acidity and the presence of antibiotics such as tetracycline [64].

The single-cell nanocoating technique can be used to functionalize probiotic cells for addressing more challenging problems. For example, spores of *Bacillus coagulans* have been nanocoated with polydopamine and chitosan and combined with calcium peroxide (CaO_2_) particles to create a spatiotemporal co-delivery system for probiotics and oxygen. This approach aims to restore the balance of intestinal microbiota and alleviate intestinal hypoxia, both of which are linked to viral pneumonia [69]. Results show that this coating protects probiotics from the acidic environment and facilitates their passage through the mucosal barrier. The sustained release of oxygen paralleled bacterial proliferation, promoting the restoration of beneficial microbiota (e.g., *Lactobacillus*, *Roseburia*) and increasing the production of short-chain fatty acids. These outcomes led to robust antiviral responses. This included reduced inflammation, a lower viral load, and decreased lung damage via the gut–lung axis [69].

Single-cell coating technology can be utilized for the concurrent delivery of probiotics alongside therapeutic drugs. For example, Peng et al. [70] exploited the inherent negative charge of probiotic *Escherichia coli* Nissle 1917 cells to bind Ca^2+^, subsequently cross-linking these cells with alginate through ionic gelation. The bacterial cells were encapsulated within a uniform layer of approximately 250 nm, effectively acting as a barrier against the acidic gastric environment while selectively disintegrating at neutral pH, thus releasing their contents in the intestine. This strategy also enabled the simultaneous co-encapsulation of the therapeutic agent 5-aminosalicylic acid within the alginate network. Proof-of-concept experiments demonstrated efficacy in treating colitis, evidenced by reduced weight loss, preservation of colon length, and improved clinical scores. Moreover, there was a significant reduction in inflammatory markers, restoration of epithelial junction proteins, and a beneficial effect on the diversity and composition of intestinal microbiota [70].

Another strategy for nanocoating probiotic cells involves incorporating them into nanoscale fibers made through the electrospinning process. Electrospinning is a technique that utilizes a strong electric field generated by applying a high voltage between the collector and the electrospinning solution. This electric force surpasses surface tension, resulting in the formation of a polymer jet propelled toward the collector [71]. Typically, in the electrospinning process, probiotic cells are directly mixed with the polymer solution. Fibers loaded with probiotics exhibit various structures, including strings, beads, or spindle-like formations, which are primarily attributed to the large volume occupied by the probiotic cells (Figure 3g,h). Research has demonstrated that the viability of probiotics is not affected by the high voltage used during electrospinning, confirming the feasibility of this processing method [72].

Common synthetic polymers used as wall materials in electrospinning include polyvinyl alcohol (PVA), polyethylene oxide (PEO), and polyvinylpyrrolidone (PVP). These materials often combine with other polymers, such as sodium alginate, corn starch, or biopolymers from waste materials like soluble dietary fiber from okara, to improve cell protection [65]. For instance, the interface between PVA and alginate improved the viability of *Lactobacillus paracasei* KS-199 by approximately one logarithmic unit after exposure to simulated gastric juice [66]. This strategy ensures that the minimum effective dose, typically within the range of 10^6^ to 10^9^ CFU/g, required for therapeutic efficacy effectively reaches the small intestine. The addition of encapsulated probiotic strains to kefir did not change its typical pseudoplastic flow behavior or viscoelastic properties [65].

Modifications to electrospinning protocols have been proposed to enhance both the efficiency of the encapsulation process and the stability of encapsulated probiotics. Techniques such as multilayer electrospinning and coaxial configurations have demonstrated promising outcomes. For example, a double-layered vehicle developed for *Lactobacillus plantarum* provided adequate protection for probiotic cells against exposure to simulated gastric conditions [73]. Coaxial fibers performed better in maintaining cell viability after exposure to heat and moisture treatments than encapsulation in uniaxial fibers.

Nanocoating techniques can enhance the effectiveness and survival of probiotics administered orally. The technologies referenced are capable of safeguarding probiotics against adverse conditions within the gastrointestinal tract, as well as during processing and storage. Nevertheless, substantial challenges persist, including scaling up for industrial application and addressing potential regulatory hurdles. Furthermore, it is essential to deepen the understanding of consumer perceptions of food and the interactions of encapsulated systems with various food types and materials; this requires further investigation. Consequently, future research should emphasize more rigorous methodologies, such as clinical trials, to substantiate the efficacy of these technologies.

### 4.2. Nanoencapsulation of Prebiotics

The incorporation of prebiotics into the diet and oral supplementation has been proven to have beneficial effects on the intestine and a positive impact on human health [74]. Prebiotics directly affect the intestine by increasing microbiota diversity through the selective promotion of beneficial bacteria’s growth and activity, while decreasing that of harmful ones [75].

Prebiotics, nondigestible non-starch polysaccharides and oligosaccharides, such as fructooligosaccharides (FOS), galactooligosaccharides (GOS), Xylooligosaccharides (XOS), Chitooligosaccharides (COS), Pectic oligosaccharides (POS), lactulose, resistant starch, inulin, pectin, and others, are dietary components that stimulate the proliferation of beneficial intestinal bacteria [75,76]. Additionally, phenolic compounds, particularly flavonoids and phenolic acids, have been demonstrated to have prebiotic effects on the intestine [77].

**Figure 4:**
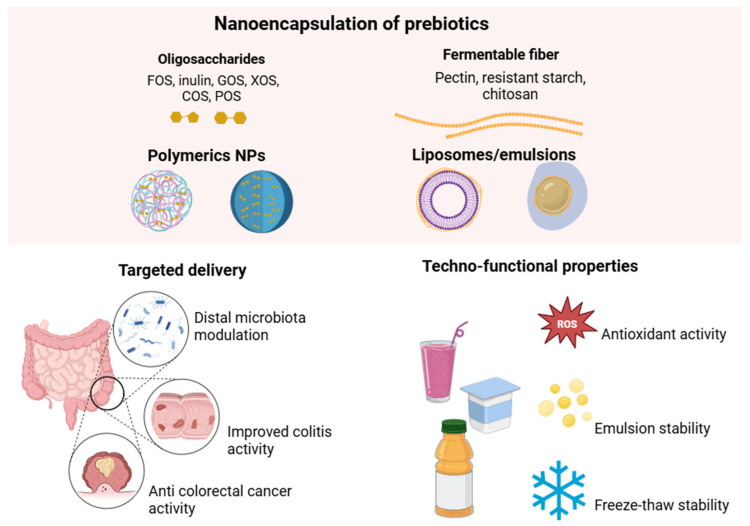
Schematic representation of the benefits of prebiotic nanoencapsulation using polymeric nanoparticles and liposomes/emulsions, including targeted delivery in the gastrointestinal tract, modulation of the microbiota, anti-inflammatory and anticancer effects, as well as improved techno-functional properties such as antioxidant activity, emulsion stability, and freeze–thaw resistance.

However, even with their potential effects, the effectiveness of prebiotics can be significantly influenced by environmental conditions during processing, storage, and movement through the gut. This can limit their benefits and therapeutic use [78]. Additionally, the loss of bioactivity during absorption in the gastrointestinal tract and the poor distribution of these active ingredients in the intestines can lead to insufficient delivery to the colon [79]. To overcome these limitations, nanoencapsulation has emerged as a promising method for improving molecular stability, targeted delivery, dose control, and protecting the functional performance of prebiotics in the intestinal tract (Figure 4) [80].

Nanostructured prebiotics, known as nano-prebiotics, can be protected during digestion, ensuring their safe release at the site of action, such as the most distal portions of the intestine, which stimulates bacterial diversity and the production of metabolites with potentially beneficial effects. The gut microbiota influences various health issues, including allergies, ulcerative colitis, and certain types of cancer. Modulating it by decreasing pathogens and increasing beneficial organisms is proven effective in treatment [76]. Nanoencapsulation of prebiotics offers a viable approach to administering isolated and combined prebiotics, thereby enhancing microbiota modulation and promoting diverse bacterial growth [57].

Modulation of the gut microbiota has become a key therapeutic target for the intervention of colonic diseases. In this context, Ren et al. [81] developed a review that emphasized the need for a deeper understanding of microbiota interactions and the benefits of identifying effective methods to deliver these bioactives locally. Prebiotics must not only achieve colon-specific delivery but also maintain intestinal homeostasis by preserving their bioactivity. The systematic review of prebiotic-based nanoparticles designed for colonic drug delivery highlights their emerging role in the therapeutic management of colonic diseases, including colitis and colorectal cancer [81]. The study details several nanoparticle systems composed exclusively of prebiotics, emphasizing their potential for targeted delivery to the colon. These systems demonstrate the ability to modulate the gut microbiota, maintaining intestinal homeostasis, and enhancing therapeutic efficacy. Prebiotic-based colonic drug delivery systems represent a novel strategy for precise, microbiota-responsive interventions targeting the treatment of inflammatory and neoplastic intestinal conditions. Dangi et al. [57] also indicate, in their extensive review, that nano-prebiotics can be incorporated into functional foods, and encapsulation leads to increased bioavailability, controlled release, and enhanced beneficial effects of these compounds. The review also addresses food safety and environmental issues [57].

Nano-prebiotics may offer a new, low-cost, accessible, and practical approach to confer health benefits to individuals, particularly as an adjunctive therapy to support patients in achieving better clinical outcomes. The controlled release and specific functional characteristics of nanomaterials are influenced by their physical, chemical, biological, and mechanical properties. Biomaterials that can be used include those of natural origin (e.g., starch, cellulose, pectin, chitosan, some proteins, and lipids) or synthetically prepared (e.g., metal-based polymers). However, nanomaterials must be biocompatible, biodegradable, and recognized as safe for use and incorporation into food [57,82].

An effective nanoencapsulation-based prebiotic delivery system is an oral therapy with anticancer properties. For instance, the encapsulation of particular disaccharides may have an effective anticancer action. A study demonstrated a novel nanoencapsulation system based on the formation of nanocomplexes between pectin and lysozyme, developed as an oral vehicle for delivering β-lactose, a natural inhibitor of galectin-3, a protein associated with colorectal cancer [83]. The encapsulation of β-lactose was effective in preventing rapid degradation in the intestine, increasing its stability, bioaccessibility, and bioavailability. The formed nanoparticles were spherical, approximately 80 nm in diameter, had a high encapsulation efficiency (>96%), a negative surface charge (−30 mV), and a homogeneous distribution (PDI < 0.2). Spectroscopic and thermal analyses revealed that electrostatic interactions and hydrogen bonds were the driving forces, indicating the encapsulation of β-lactose. Analysis of colorectal cancer cells revealed that the nanoparticles were incorporated in a time-dependent manner, suggesting their potential use as an oral delivery system for bioactive compounds in food matrices [83].

A recent study investigated the effect of incorporating four prebiotic saccharides: gum arabic (GA), fructooligosaccharide (FOS), konjac glucomannan (KGM), and inulin (INU) into urolithin A-loaded liposomes (UroA-LPs) [84]. The developed formulation was evaluated for parameters such as encapsulation efficiency, physicochemical characteristics, and stability in an in vitro digestion model. The effects on intestinal microbiota were also investigated through in vitro colonic fermentation. The results indicated that the GA-coated liposomes had high encapsulation efficiency, bioaccessibility, and thermal stability, with bioaccessibility being approximately twice that of UroA-LPs. The FOS-coated UroA-LPs showed the best freeze–thaw stability. The addition of saccharides notably increased the abundance of *Bacteroidota* and reduced that of *Proteobacteria* and *Actinobacteria*. UroA-LPs coated with FOS, INU, and GA showed the highest abundance of beneficial bacteria, such as *Parabacteroides*, *Monoglobus*, and *Phascolarctobacterium*, respectively. FOS increased the levels of acetic acid, butyric acid, and isobutyric acid. Prebiotic saccharides can enhance the encapsulation efficiency, physicochemical stability, and regulation of gut microbiota, while promoting the bioaccessibility of UroA, which may lay the foundation for its application in the food industry.

A study developed by Feng et al. [85] utilized *Ramulus mori* polysaccharides encapsulated in Poly(lactic-co-glycolic acid) (PLGA) to investigate the anti-inflammatory effects of these thin, dried mulberry branches, which possess potent anti-inflammatory, antidiabetic, and antioxidant properties. The diameter of the nanoparticles was 205.6 ± 1.86 nm. Regarding the biological effects of these nanoparticles in mice with colitis (by DSS oral administration), attenuation of body weight loss and restoration of colon length were observed, as well as reduced production of IFN-γ and IL-6, improved IL-10, and increased production of short-chain fatty acids, such as acetate, propionate, and butyrate, in the colon with colitis. Another critical finding was the protection against a reduction in gut microbiota diversity caused by inflammation, as well as positive changes in the ratio of Firmicutes to Bacteroidetes. The nanoformulation identified in this study suggests the potential of nanoparticles in mitigating metabolic disturbances associated with colonic inflammation, indicating that they could serve as a potential treatment for prebiotics.

A study investigated the production and potential application of nanoencapsulated chito-oligosaccharides in foods [86]. The chitooligosaccharide was produced by enzymatic hydrolysis of chitosan (chitosanase from *Bacillus cereus*). Prebiotic activity, antioxidant activity, and stability were evaluated when the compound was included in food (yogurt). The results indicated that the chitooligosaccharide produced was water-soluble (MW 2.005 kDa) and presented a high degree of acetylation. Regarding bioactivity, potent prebiotic and antioxidant properties, as well as stability, were observed. In turn, the encapsulation of chitooligosaccharide in nanoparticles and microparticles aims to promote stability compared with non-encapsulated chitooligosaccharide. Nanoparticles measuring 100 nm were observed to be stable in yogurt and exhibited excellent sensory acceptance. This suggests that nanoencapsulation has the potential to be a viable inclusion in food products.

On the other hand, in addition to the properties of inulin and pectin as prebiotics, it is essential to emphasize that their multifunctional and flexible structure provides stabilization for other bioactive compounds and targeted delivery capabilities, indicating the potential of these polysaccharides as vehicles for administration as well. The molecular structure can be easily modified to increase bioavailability, improve cellular uptake, and achieve targeted, sustained, and controlled release in the intestine [87,88].

A wide variety of prebiotics remain unexplored regarding their behavior in nanostructured systems, as well as numerous possibilities for combinations with different biomaterials that can ensure safe, effective, and targeted delivery to the intestine. Also, it is necessary to conduct a study to scale up the production of nano-prebiotics, enabling their application in both the food and pharmaceutical industries [57]. Additionally, safety and toxicity issues still require further investigation. Furthermore, it is essential to investigate the use of these systems in specific pathological conditions, such as colitis and colorectal cancer, considering oral administration as an adjuvant therapeutic strategy. The interaction between encapsulated prebiotics and the host microbiota profile in in vivo models, as well as the determination of optimal doses for supplementation, is crucial for establishing nutritional guidelines. Well-designed in vivo studies are strongly recommended to validate the efficacy, safety, and clinical applicability of these innovative approaches. Corroborating in vitro studies are also needed to translate preclinical results into practical clinical applications and support the use of nano-prebiotics for industrial applications.

### 4.3. Nanoencapsulation of Synbiotics

**Figure 5:**
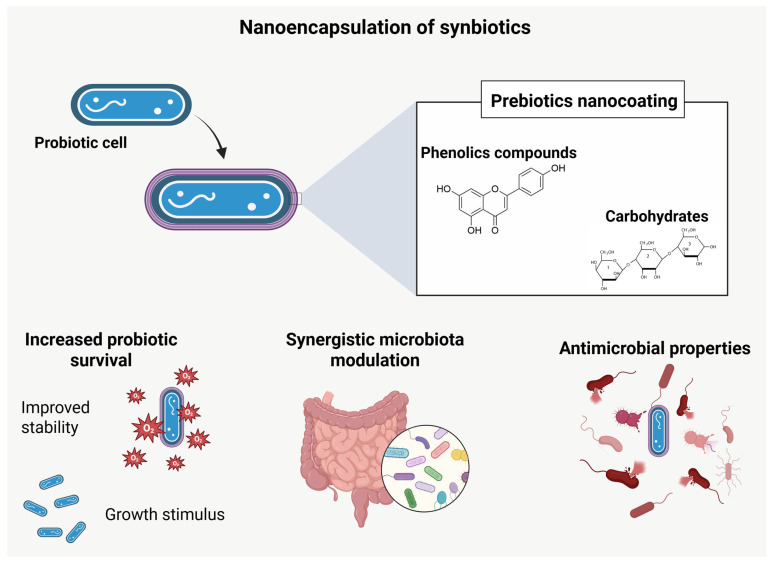
Schematic representation of synbiotic nanoencapsulation, highlighting the coating of probiotic cells with prebiotics (phenolic compounds and prebiotic carbohydrates). This approach promotes increased probiotic survival, synergistic modulation of microbiota, and enhanced antimicrobial and antioxidant properties.

The application of nanoencapsulation in synbiotics—synergistic combinations of probiotics and prebiotics—primarily aims to maximize their functional efficacy and stability in relevant matrices, such as foods, supplements, or pharmaceuticals (Figure 5). As with isolated probiotics, encapsulation methodologies for synbiotics are designed to protect and enable the controlled release of both live microorganisms and fermentable substrates, which are often vulnerable to adverse environmental conditions, including gastric acidity, bile salts, heat, oxygen, and enzymatic activity. The expertise accumulated in nanoencapsulating probiotics can be strategically used in the development of nano-synbiotics, taking advantage of the potential of prebiotics not only as specific substrates for co-administered microorganisms but also as agents capable of favorably modulating the host’s microbiota. In this way, prebiotics may contribute to increasing the viability and persistence of probiotics until they reach their target site, while also exerting synergistic effects that promote intestinal health.

Single-cell nanocoating is a highly versatile approach for integrating probiotics and prebiotics into nanoencapsulation systems. Lim et al. [89] presented a nanocoating system for *Lactobacillus plantarum* utilizing phenolic compounds and cellulose nanocrystals (CNCs) derived from green tea waste. The methodology employed a cell-mediated catalytic process, in which manganese ions secreted by the lactobacilli induced the oxidative polymerization of phenolics on the bacterial surface, forming a protective layer that was subsequently functionalized with CNCs through a “one-pot” conjugation. Importantly, phenolic compounds and CNCs as nanocoating agents can act as prebiotics, potentially classifying the entire encapsulation system as symbiotic. This system demonstrated substantial improvements in storage stability and, under simulated gastric conditions, exhibited more than a 2-log increase in survival compared to free probiotic cells. The phenolic protective barrier was primarily responsible for the enhanced survival of the probiotics, along with other factors, by limiting hydrogen ion diffusion and preserving membrane integrity.

The functionalization of probiotic cells through single-cell nanocoating is a promising approach for managing inflammatory bowel diseases. In the study by Hu et al. [90], *Escherichia coli* Nissle 1917 cells were coated with polyphenolic nanoparticles formed by the self-assembly of tannic acid and a self-polymerizable aromatic dithiol, thus combining the microbiota-modulating capability of the probiotic with the antioxidant and anti-inflammatory properties of polyphenols, which additionally exert prebiotic functions. The coating provided significant protection to the probiotic cells against the adverse conditions of the gastrointestinal tract, including gastric acidity, bile salts, an oxidative environment, and intestinal fluids. This modification resulted in a marked alleviation of clinical symptoms of colitis, including weight loss and colon shortening, as well as a reduction in inflammatory markers and the restoration of epithelial junction proteins (ZO-1 and Occludin), which are essential for the integrity of the intestinal barrier. The treatment also favorably modulated the composition of the intestinal microbiota, with an increase in the relative abundance of beneficial bacteria (such as *Akkermansia* and *Lachnospiraceae*) and a reduction in pathogenic microorganisms, including *Escherichia*-*Shigella*, thereby promoting microbial homeostasis and effectively alleviating intestinal inflammation.

The incorporation of prebiotic oligosaccharides into nanoencapsulated synbiotic systems constitutes a promising strategy to promote the colonization and proliferation of probiotic microorganisms during their release along the gastrointestinal tract. An example of this approach is the inclusion of inulin in PLGA nanoparticles integrated with *Bifidobacterium* strains encapsulated in a gum arabic and alginate matrix [91]. This synbiotic combination resulted in greater resistance of the probiotic cells during exposure to simulated digestive fluids, demonstrating a significant improvement in microbial viability under adverse conditions. The presence of the prebiotic not only provided additional physical protection but also acted as a selective substrate, enhancing the survival and functionality of the probiotics within the intestinal environment. Similar results were obtained by Noman et al. [92], who observed not only increased viability of *Lactobacillus rhamnosus* after coating with starch nanoparticles (derived from water chestnut and rice) under simulated gastric and intestinal conditions, but also increased thermal tolerance of the probiotic strains. This improvement in thermal resistance is particularly relevant for protecting probiotic strains during critical industrial processing steps, such as spray drying, pasteurization, and lyophilization, as well as for enhancing their stability during the storage of pharmaceutical products.

The antimicrobial properties of nanoencapsulated synbiotics demonstrate promising potential for application within the livestock production chain, particularly as an alternative to conventional antibiotics. This approach may help reduce the selective pressure exerted by the indiscriminate use of antimicrobials, thereby aiding in the mitigation of the emergence and spread of resistant bacterial strains. An example of this strategy was demonstrated by combining phytogenic compounds extracted from pomegranate peel with multi-species probiotics (*Lactococcus lactis*, *Lactobacillus plantarum*, *Lactobacillus paracasei,* and *Saccharomyces cerevisiae*), encapsulated in sodium alginate and CaCl_2_ nanocapsules [93]. This formulation exhibited high inhibitory capacity against relevant pathogens, including *Escherichia coli* (ATCC 8739), *Staphylococcus aureus* (ATCC 6538), *Pseudomonas aeruginosa* (ATCC 90274), as well as the fungi *Aspergillus niger* and *Aspergillus flavus*. The observed antimicrobial effects were attributed to the increased viability of the encapsulated probiotics, the controlled release of bioactive compounds, and the greater contact area provided by the nanostructure, highlighting the efficacy of the formulation in microbial control with potential application in animal production systems.

The use of such synbiotic nanoparticles may represent a promising approach for mitigating damage caused by food-borne toxins. Swetha Kumar and Sahabudeen Mohideen [94] demonstrated that chitosan-coated probiotic nanoparticles (*Lactobacillus fermentum*) can be used to mitigate acrylamide-induced toxicity in an animal model (*Drosophila melanogaster*). Flies exposed to acrylamide and simultaneously treated with the nanoencapsulated synbiotics showed significant improvement in survival and behavioral parameters, clearly demonstrating a protective effect against acrylamide toxicity. The nanoparticles were effective in normalizing levels of free radicals and restoring the activity of antioxidant enzymes that had been altered due to exposure to the toxin. Furthermore, the nanoparticles protected against mitochondrial membrane depolarization in the ovaries of female flies exposed to acrylamide, indicating potential prevention of cellular damage and apoptosis.

Recent advances in individual cell coatings and the strategic integration of prebiotic compounds have demonstrated not only improved survival of probiotic cells but also enhanced therapeutic benefits, such as effective modulation of the intestinal microbiota, antioxidant and anti-inflammatory properties, as well as protection against foodborne toxins and pathogens. Similarly to probiotics and prebiotics, there is still a lack of research regarding the scalability and optimization of processes to ensure the economic viability and commercial acceptance of nanoencapsulated synbiotics.

### 4.4. Nanoencapsulation of Postbiotics

**Figure 6:**
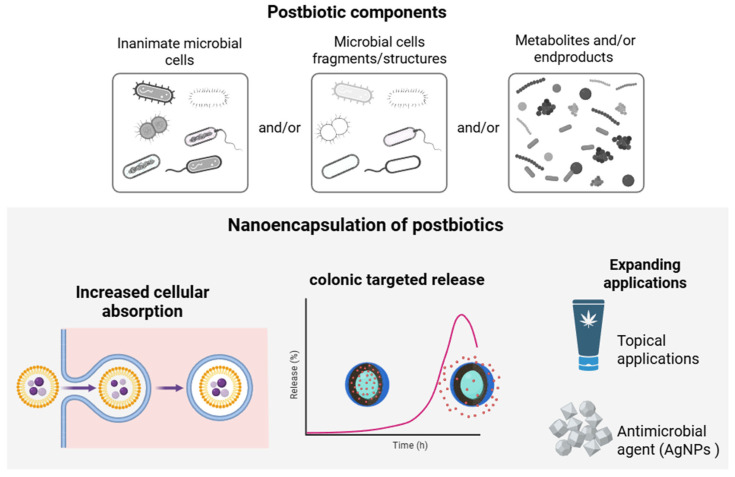
Schematic representation of postbiotic components (inanimate microbial cells, fragments, or metabolites) and the benefits of nanoencapsulation, including increased cellular absorption, targeted colonic release, and expanded applications such as topical use and as a reducing agent in the fabrication of antimicrobial silver nanoparticles.

The nanoencapsulation of postbiotics enables enhanced stability, bioavailability, and efficacy of bioactive compounds derived from probiotic microorganisms. Unlike synbiotics or live probiotics, postbiotics—non-viable bioactive substances produced by microorganisms—offer several important advantages, including greater thermal stability, microbiological safety, and the absence of risks associated with bacterial translocation. The application of nanocarriers further potentiates these characteristics by protecting the compounds from degradation and promoting their targeted release and improved absorption (Figure 6) [95].

Postbiotic-loaded nanoparticles tend to produce more reproducible effects, as they do not depend on the viability of probiotic cells. The beneficial effects observed combine mechanisms of microbial, antioxidant, and immunomodulatory activity [96]. Nevertheless, there is still little standardization regarding production processes, long-term toxicological evaluation, and the regulation of these systems’ use in humans and animals [95].

The use of nanocapsules allows for the protection of postbiotic bioactive compounds against adverse environmental conditions, including variations in pH, temperature, enzymatic activity, and chemical degradation. Such protection significantly enhances therapeutic potential by enabling controlled and site-specific release in target biological locations. The development of arginine-chitosan and fucoidan-based nanoparticles for encapsulating the postbiotic derived from *Lacticaseibacillus paracasei* GMNL-133 (SGMNL-133) demonstrated high efficacy in protecting the bioactive compounds from gastric acid degradation [97]. In addition to increased stability, a significant enhancement in the intracellular penetration of SGMNL-133 was observed, thereby potentiating its interaction with gastric tumor cells. In vivo assays confirmed that the nanoencapsulated system showed superior results in reducing tumor growth and attenuating tissue inflammatory processes compared to the free postbiotic, highlighting the therapeutic potential of this nanotechnological approach.

Stabilization of intestinal barrier integrity is a primary objective in the use of postbiotics. Yu et al. [98] proposed nanoparticles designed for colonic delivery of butyric acid, utilizing a polyvinyl butyrate polymeric core system coated with shellac resin. This structure enabled adequate protection against gastric degradation and sustained release in the colon. As a result, a significant suppression of macrophage inflammatory activation, regulation of redox balance, and favorable remodeling of the intestinal microbiota were observed. The accumulation of butyric acid led to a significant restoration of intestinal epithelial barrier integrity and a reduction in systemic inflammation, which, in turn, reestablished the balance between osteoblastic and osteoclastic activity. The study highlights the gut–bone axis as a strategic pathway for treating osteoporosis related to inflammatory bowel disease, as well as postmenopausal osteoporosis.

The use of nanoencapsulation technology further enhances the versatility of postbiotic applications. For example, Gökçe and Aslan (2024) [99] employed liposomal gel formulations containing next-generation postbiotics, yielding satisfactory results in terms of controlled release, a rheological profile suitable for topical use, and preservation of the original antimicrobial activities. Similarly, bacteriocins derived from *Enterococcus* were encapsulated in liposomes, resulting in a fourfold increase in their antimicrobial activity against vancomycin-resistant *Enterococcus faecalis* V853 [100]. This encapsulation not only preserved the integrity of the molecules but also enhanced their therapeutic efficacy.

The biosynthesis of silver nanoparticles (AgNPs) using postbiotics is a promising approach for producing antimicrobial agents. Postbiotics are employed as reducing agents, and this method offers advantages over conventional techniques due to their environmentally friendly nature, biocompatibility, and stability. Films made from carboxymethyl chitosan were integrated with silver nanoparticles anchored on covalent organic frameworks (COFs), which provided adequate protection against microbial contamination (*Staphylococcus aureus*, *Escherichia coli*, *Bacillus cereus*, *Cronobacter sakazakii*, and *Listeria monocytogenes*), especially in citrus fruits, thereby prolonging product shelf life, mitigating post-harvest losses, and significantly improving the mechanical strength of the films [101]. A postbiotic product from *Ligilactobacillus salivarius* KC27L was used for the biogenic synthesis of AgNPs, which demonstrated effective antimicrobial activity against various relevant pathogens, such as *Escherichia coli*, *Pseudomonas aeruginosa*, and *Streptococcus mutans* [102]. Additionally, the AgNPs exhibited notable antioxidant and anti-biofilm activities.

The application of nanocarrier systems enhances the properties of postbiotics by providing adequate protection against chemical and enzymatic degradation, as well as adverse gastrointestinal conditions, while also enabling controlled and site-specific release. Despite these advantages, substantial challenges still need to be addressed. Among these, the need for rigorous standardization of processes and long-term toxicological safety of postbiotics is highlighted. These factors are crucial for ensuring the regulatory and commercial approval of such products.

## 5. Conclusions and Outlook

Nanoencapsulation strategies possess the potential to fundamentally transform the delivery and efficacy of biotics within the gastrointestinal tract. By facilitating the incorporation of novel ingredients and broadening the spectrum of possible applications, these methodologies are attracting attention from healthcare professionals, academic researchers, and the food and pharmaceutical industries. This growing interest is driven not only by the substantial health benefits associated with these systems but also by their innovative character. Advances in this domain thus delineate a promising frontier for the development of functional solutions that aim to enhance human health and nutrition.

Nevertheless, several critical challenges persist, including the need for standardized manufacturing processes, addressing regulatory and industrial scalability issues, and ensuring the stability and safety of nanoencapsulated systems. Furthermore, production costs and the comprehensive assessment of long-term toxicological risks continue to hinder the widespread adoption of these technologies in practical applications. The scarcity of robust, well-designed, and standardized clinical trials further restricts the establishment of reliable and evidence-based recommendations for the clinical use of these emerging systems.

Future research efforts should prioritize interpreting the complex interactions between nanostructured delivery systems and the host’s gut microbiota profile, as well as optimizing nanoencapsulation methodologies for enhanced efficiency and scalability. Additionally, investigating novel combinations of biotics and biomaterials and systematically evaluating their effects in diverse pathological contexts is an essential step toward consolidating the therapeutic and preventive potential of these technologies in maintaining intestinal, and consequently, overall health.

## Data Availability

Not applicable.
